# Risk factors for the development of severe breast cancer-related lymphedema: a retrospective cohort study

**DOI:** 10.1186/s12885-023-10814-5

**Published:** 2023-04-20

**Authors:** Xiaozhen Liu, Kewang Sun, Hongjian Yang, Lingli Xia, Kefeng Lu, Xuli Meng, Yongfeng Li

**Affiliations:** 1 General Surgery, Cancer center, Department of Breast Surgery, Zhejiang Provincial People’s Hospital (Affiliated People’s Hospital, Hangzhou Medical College), Hangzhou, 310014 Zhejiang China; 2 Department of Breast Surgery, Institute of Cancer Research and Basic Medical Sciences of Chinese Academy of Sciences (Zhejiang Cancer Hospital), Hangzhou, 310022 Zhejiang China; 3 Department of outpatient service, Institute of Cancer Research and Basic Medical Sciences of Chinese Academy of Sciences (Zhejiang Cancer Hospital), Hangzhou, 310022 Zhejiang China; 4 Cancer center, Department of Ultrasound Medicine, Affiliated People’s Hospital, Zhejiang Provincial People’s Hospital, Hangzhou Medical college), Hangzhou, 310014 Zhejiang China

**Keywords:** Lymphedema, Nomogram, Lymph node, Chemotherapy, Radiotherapy

## Abstract

**Background:**

Severe lymphedema presents a challenge in terms of treatment due to the significant formation of scar tissue that accompanies it. The aim of this study was to identify intraoperative and preoperative risk factors of severe lymphedema and to develop a nomogram for estimating the risk of severe lymphedema within 3 years of surgery.

**Method:**

Data was collected from a retrospective cohort of 326 patients with BCRL at the Zhejiang Cancer Hospital from November 2015 to November 2018. Univariate and multivariate logistic regression analysis was conducted to identify predictive indicators of severe lymphedema. A nomogram was developed to further improve the clinical applicability.

**Results:**

In the retrospective cohort, the ratio of severe/non-severe lymphedema within 3 years of surgery was 1:3. Independent risk factors for severe lymphedema were determined to be age, positive lymph nodes, interpectoral (Rotter’s) lymph nodes (IPNs) dissection, and educational level. IPNs dissection was found to contribute greatly to the development of severe lymphedema with a higher odds ratio (7.76; 95% CI: 3.87–15.54) than other risk factors. A nomogram was developed by integrating age, positive lymph nodes, IPNs dissection, and educational level, which yielded a C-index of 0.810 and 0.681 in the training and validation cohort, respectively. This suggested a moderate performance of the nomogram in predicting the risk of severe lymphedema within 3 years of surgery. The cut-off values of the low-, medium- and high-risk probabilities were 0.0876 and 0.3498, and the severe lymphedema exhibited a significantly higher risk probability as compared with the non-severe lymphedema.

**Conclusion:**

This study identified the risk factors of severe lymphedema and highlighted the substantial contribution of IPNs dissection to the severity of lymphedema.

## Introduction

Lymphedema is a condition caused by impairment of the lymphatic system, which usually results in progressive swelling due to an abnormal accumulation of lymph fluid [1, 2]. Sufferers can experience negative physical, psychological, and emotional effects, and incur additional financial burdens [3]. Breast cancer-related lymphedema (BCRL) can be a painful, potential debilitating complication after axillary dissection and regional nodal irradiation for breast cancer, having a rate ranging from 9 to 65% [4–6]. But due to the wider use of sentinel lymph node biopsy, the incidence of BCRL has decreased significantly, estimated to be 1–7% [7].

Although secondary lymphedema symptoms are usually mild and temporary, this group is three times more likely to suffer from moderate or severe edema compared to those with no symptoms[8]. The treatment of mild to moderate secondary lymphedema symptoms involves manual lymphatic drainage, massage, compression garments, and physical therapy [9]. However, for patients with advanced lymphedema, few treatments, including surgery, can halt the progress of the condition. Despite being the standard treatment for breast cancer, axillary lymph node dissection (ALND) may result in lymphedema in 20–50% of patients [10]. Other factors influencing include modified radical mastectomy (MRM), radiotherapy, body mass index (BMI), cellulitis, hypertension, education level, and chemotherapy[5, 11–18].

To date, no systematic evaluation of severe lymphedema risk factors has been conducted. In this study, we conducted a retrospective clinical analysis of BCRL patients who underwent either conservative breast cancer surgery or mastectomy, with the ami of pinpointing the risk factors that are likely to lead to severe lymphedema. A nomogram was further developed to facilitate the prediction of severe lymphedema risk in future patients.

## Methods and materials

### Study design and patients

This retrospective analysis was conducted in compliance with the tenets of the Declaration of Helsinki, and the study protocol was approved by the Ethics Committee of Zhejiang Cancer Hospital. The Ethics committee of Zhejiang Cancer Hospital waived the need for written informed consent due to the retrospective nature of the study. The electronic report database of Zhejiang Cancer Hospital was searched for patients with BCRL who underwent conservative breast cancer surgery or mastectomy from November 2015 to November 2018. Patients with a 3-year period from operation time were enrolled in the study, while for those with multiple diagnostic records, the most severe degree of edema was adopted as their final outcome. The whole cohort were randomly divided into the training cohort (228) and validation cohort (98) with a ratio of 7:3.

### Inclusion and exclusion criteria

Inclusion criteria consist of female participants aged 18 to 75 years old who have been diagnosed with lymphoedema within 3 years of having undergone breast cancer surgery. Exclusion criteria include patients with recurrent or metastatic breast cancer; a history of upper limb surgery or trauma; systemic diseases known to cause swelling (e.g. myocardial infarction, renal dysfunction, gastrointestinal diseases); pregnancy or lactation; and prior treatment for arm lymphoedema.

### Predictor selection

Only those potential predictors with prior clinical knowledge were selected for consideration[19–23]. These were identified from the published literature and clinical reasoning and included age, tumor size, circle of chemotherapy, serum cholesterol, BMI, estrogen receptor (ER), progesterone receptor (PR), human epidermal growth factor receptor 2 (HER2), Ki-67, neoadjuvant chemotherapy, docetaxel, chemotherapy, radiotherapy, education level, hypertension, and ALND. However, all the participants in this study underwent level I/II ALND, making it impossible to gauge the role ALND played in severe lymphedema. Hence, we opted to use the data of the number of positive lymph nodes and total lymph node dissection, and interpectoral lymph nodes (IPNs) dissection in order to accurately and spatially reflect the damage to the patient’s lymphatic system and identify the association between them and the risk of severe lymphedema.

### Identification of severe lymphedema

Lymphedema was defined as symptomatic arm swelling with a difference of 2 cm or more the circumference at two adjacent points between the affected and contralateral arms[14]. Specifically, circumference of both arms is measured 5 cm apart from each other with reference to the elbow flexion. The maximum value of the difference in arm circumference is used as the diagnostic result. Non-severe and severe arm lymphedema was defined as having a circumference difference of 2 to 4 cm and more than 4 cm, respectively, in the the forearm at any measurement site.

### Statistical analysis

All statistical analyses were executed using SPSS version 25.0 software (IBM, Armonk, USA) and R software version 4.2.0 (http://www.Rproject.org). A binary logistic regression model was applied to identify and examine risk factors for severe lymphoedema. A forest plot was employed to display the odds ratios (ORs) (95% confidence interval [CI]) and p values of each variable. The candidate variables associated with severe lymphoedema with significance (p values < 0.05) were tested in a multivariable logistic regression model in the training cohort. A nomogram incorporating risk factors was created to predict the likelihood of severe BCRL and validated internally by bootstrapping and externally in the validation cohort. The calibration curve was plotted to observe the predictive performance of the nomogram. Patients from the retrospective cohort were divided into three risk groups (low, medium, and high risk) based on the first and third quartile of probabilities from the nomogram model. An unpaired t-test was used to compare the risk scores between the non-severe patients and severe patients. All p-values were two-sided and p-value < 0.05 was regarded as significant.

## Results

### Characteristics of the eligible patients

This retrospective cohort enrolled 326 patients with BCRL, with a median (interquartile range) age of 48 (43–57) years. The median (interquartile range) tumor size, positive lymph nodes, total lymph node dissection, serum cholesterol, and BMI were 20 (5–30), 2(0–4), 18 (15–22), 5.107 (4.315–5.869), and 24.53 (22.43–26.86), respectively. IPNs dissection was performed in 18.4% (60/326) of the patients. Most of patients (92.3%) underwent chemotherapy, while 71.8% received radiotherapy, The median (interquartile range) circle of chemotherapy was 6 (4–8). Docetaxel was administered to 278 (85.3%) patients, while only 32.5% received neoadjuvant chemotherapy. In addition, 62.6% of the patients had a high educational level. In terms of marker genes, the expressions of ER, PR, Ki-67 and HER-2 were 66.3%, 56.1%, 50.3%, and 30%, respectively. Based on the diagnostic criteria, 235 and 91 patients were assigned to the non-severe lymphedema and severe lymphedema cohort, respectively. The initial characteristics of the cohort are presented in Table 1.

**Table 1:** Characteristics of BCRL patients in the retrospective cohort

	All patients	Non-severe patients	Severe patients	*p* value
Subjects	326	235	91	
Age^#^	48(43–57)	47(43–55)	52(44–61)	0.018
Tumor size^#^	20(5–30)	20(5–30)	23(6–35)	0.214
Positive lymph nodes^#^	2(0–4)	2(0–3)	0(0–1)	< 0.001
Total lymph node dissection^#^	18(15–22)	18(15–22)	19(16–22)	0.465
Cycles^#^	6(4–8)	6(4–8)	6(6–8)	0.664
Serum cholesterol^#^	5.107(4.315–5.869)	5.104(4.268–5.830)	5.126(4.389–6.040)	0.216
BMI^#^	24.53(22.43–26.86)	24.41(22.27–26.75)	24.77(22.77–27.24)	0.492
IPNs dissection^*^				< 0.001
Yes	60 (18.4)	18 (7.7)	42 (46.2)	
No	266 (81.6)	217 (92.3)	49 (53.8)	
ER^*^				0.480
Positive	216 (66.3)	153 (65.1)	63 (69.2)	
Negative	110 (33.7)	82 (34.9)	28 (30.8)	
PR^*^				0.085
Positive	183 (56.1)	125 (53.2)	58 (63.7)	
Negative	143 (43.9)	110 (46.8)	33 (36.3)	
Ki-67^*^				0.493
Positive	164 (50.3)	121 (51.5)	43 (47.3)	
Negative	162 (49.7)	114 (48.5)	48 (52.7)	
HER2^*^				0.945
Positive	112 (34.4)	81 (34.5)	31 (34.1)	
Negative	214 (65.6)	154 (65.5)	60 (65.9)	
Neoadjuvant chemotherapy^*^				0.914
Yes	106 (32.5)	76 (32.3)	30 (33.0)	
No	220 (67.5)	159 (67.7)	61 (67.0)	
Docetaxel^*^				0.125
Yes	278 (85.3)	196 (83.4)	82 (90.1)	
No	48 (14.7)	39 (16.6)	9 (9.9)	
Chemotherapy^*^				0.359
Yes	301 (92.3)	215 (91.5)	86 (94.5)	
No	25 (7.7)	20 (8.5)	5 (5.5)	
Radiotherapy^*^				0.017
Yes	234 (71.8)	160 (68.1)	74 (81.3)	
No	92 (28.2)	75 (31.9)	17 (18.7)	
Education level^*^				< 0.001
High-Bachelor’s degree or more	204 (62.6)	170 (72.3)	34 (37.4)	
Low-Associates degree or less	122 (37.4)	65 (27.7)	57 (62.6)	
Hypertension^*^				0.079
Yes	56 (17.2)	35 (14.9)	21 (23.1)	
No	270 (82.8)	200 (85.1)	70 (76.9)	

Abbreviations: BMI: Body Mass Index, IPNs: interpectoral lymph nodes, ER: estrogen receptor, PR: progesterone receptor, HER2: human epidermal growth factor receptor 2. ^#^ Expressed as median (interquartile range), ^*^Data are presented as numbers with percentages in parentheses

### Risk factors for severe lymphedema

**Fig. 1:**
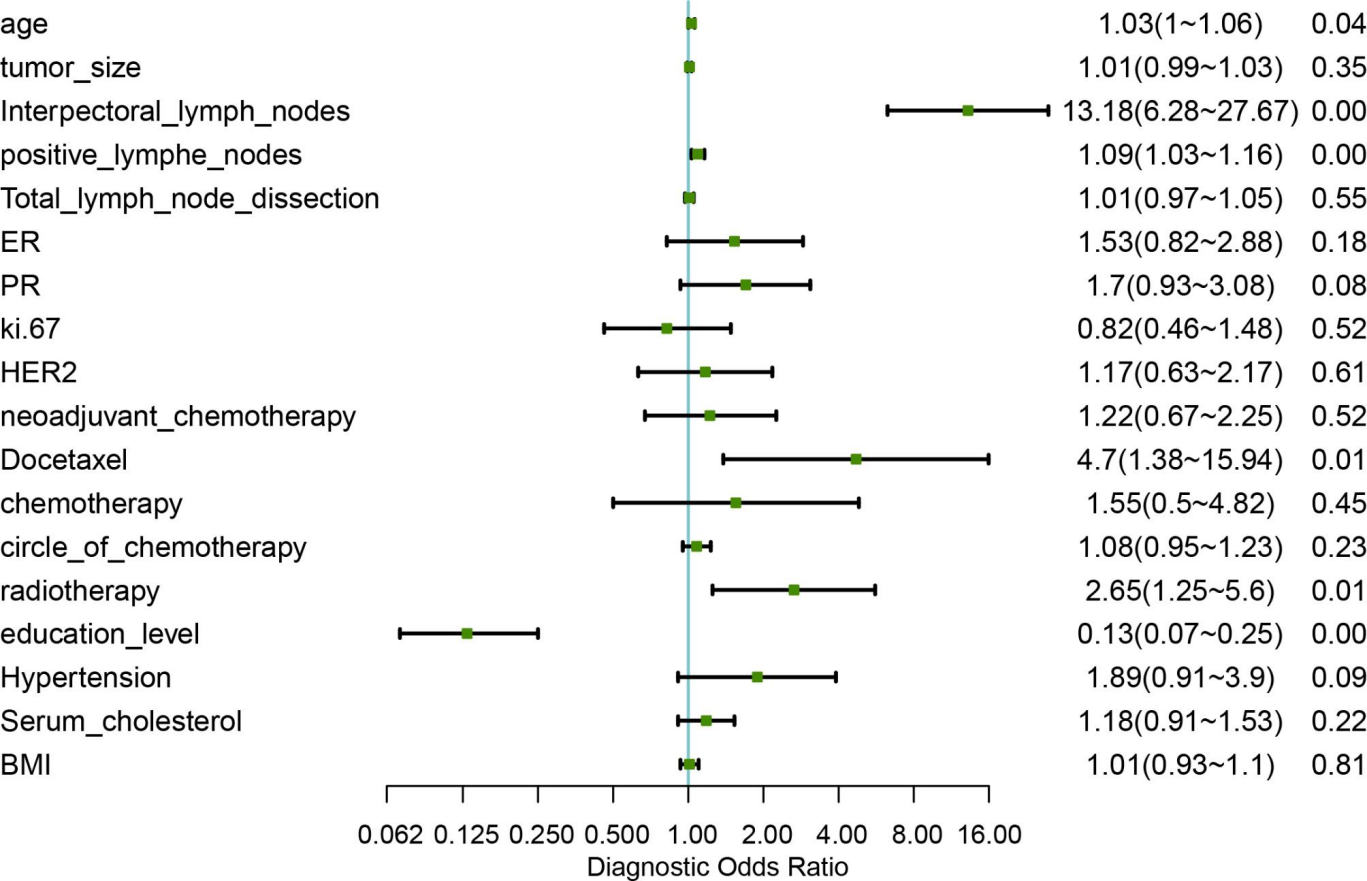
The forestplot of the odd ratios (OR) of each variables for severe lyphedema

A univariate logistic regression analysis was performed to identify predictive indicators associated with severe lymphedema, and the forestplot was generated to illustrate the OR and P-value of each variable (Fig. 1). Results revealed that age, IPNs dissection, positive lymph nodes, docetaxel treatment, radiotherapy, and education level were significantly associated with the severity of lymphedema (p < 0.05). Multivariable analysis suggested that all predictive factors (p < 0.05) from univariate analysis were significantly associated with the severity of lymphedema (Table 2), except for radiotherapy. Specifically, significant predictors were age (OR, 1.04; 95% CI, 1.01–1.07; p = 0.02), IPNs dissection (OR, 7.76; 95% CI, 3.87–15.54; p < 0.01), positive lymph nodes (OR, 1.06; 95% CI, 1.00−1.13, p = 0.03994), and education level (OR, 0.41; 95% CI, 0.23–0.73; p < 0.01).

**Table 2:** Remaining predictors of the nomogram model after backward elimination for the prediction of severity of BCRL

Factor	Nomogram model OR (95% CI)	*p* Value
Age	1.04 (1.01–1.07)	0.02
IPNs dissection	7.76 (3.87–15.54)	< 0.01
Positive lymphe nodes	1.06 (1.00−1.13)	0.04
Radiotherapy	1.79 (0.90–3.55)	0.10
Education level	0.41(0.23–0.73)	< 0.01
Docetaxel	1.36 (0.56–3.31)	0.49

### Development and validation of the BCRL nomogram

**Fig. 2:**
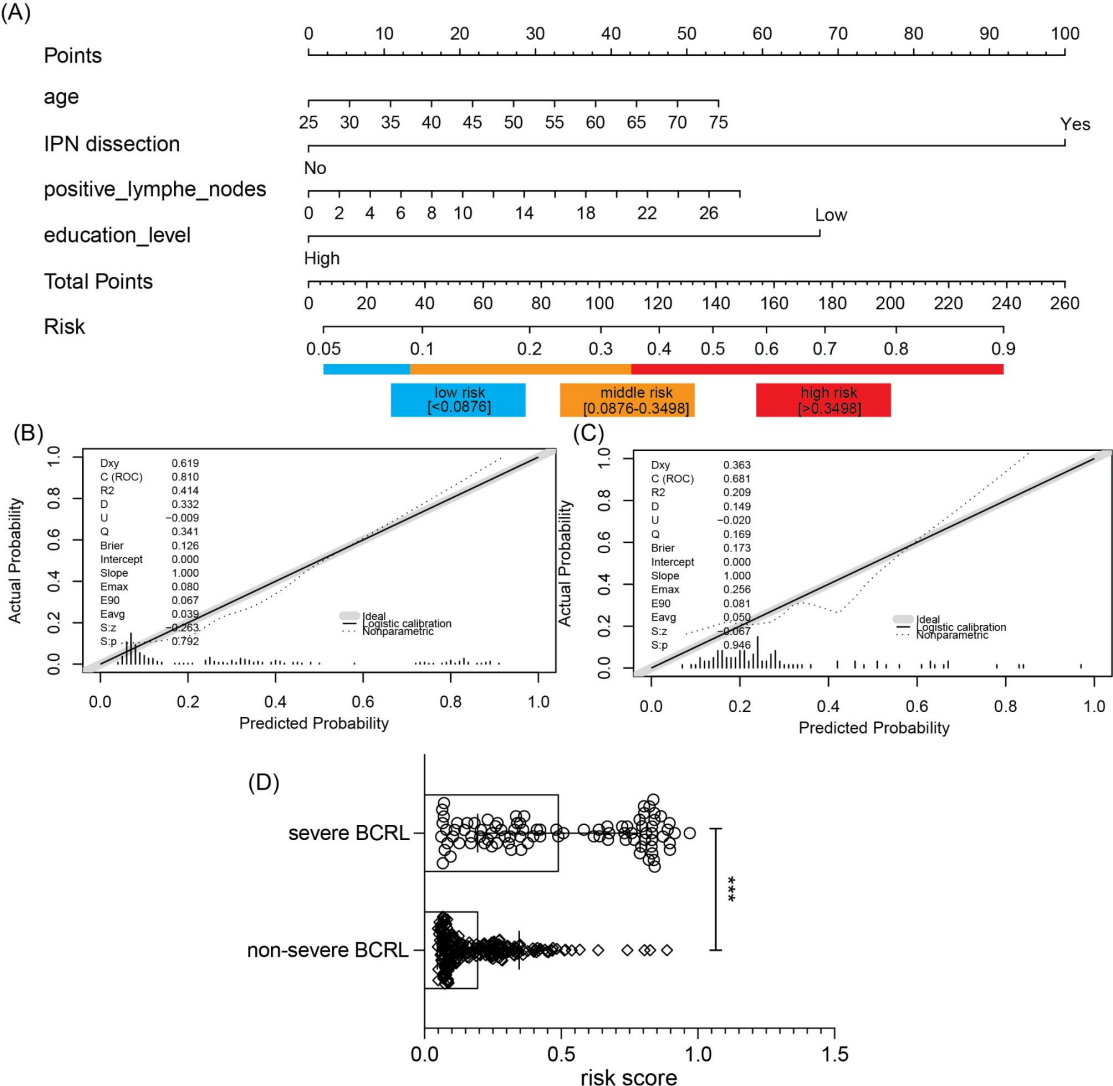
Severe-illness risk nomogram and triage tool for clinicians. (A) The nomogram integrating age, IPNs, positive lymph nodes, and education level was constructed to predict the 3-year risk probability of severe lymphedema (B) the calibration curve of internally validation with bootstrapping for the nomogram model. (C) the calibration curve for the nomogram model in the validation cohort. (D) Distribution of risk probability in the non-severe and severe lymphedema. *** p < 0.001

As shown in Fig. 2A, a nomogram was constructed by integrating age, IPNs dissection, positive lymph nodes, and education level. To reclassify the retrospective cohort, we applied two cut-off values (0.0876 and 0.3498,) to divide the cohort into the low-, medium- and high-risk probabilities groups. The model was internally evaluated through 1000 bootstrapped samples and the calibration curve displayed predicted and actual probabilities. The C-index of the calibration curve in the internal calibration was 0.810 (Fig. 2B**)**, while the C-index of the calibration curve for the validation set was 0.681 (Fig. 2C). Moreover, the risk probabilities in the severe cohort was significantly higher than that in the non-severe cohort (Fig. 2D, p < 0.001).

## Discussion

Breast cancer-related lymphedema is one of the most feared complications of post-surgery breast cancer survivors. Patients suffering from lymphedema frequently report a lower quality of life, poorer mobility, reduced self-esteem, coupled with an increased a higher risk of infection [16, 17]. Severe lymphedema is challenging to manage, has a higher risk of recurrence and progression, and requires long-term follow-up and monitoring. Identifying risk factors is crucial, as it aids physicians in recognizing high-risk patients and offering early interventions to prevent disease progression. A deeper understanding of risk factors can also lead to more effective treatment options. Despite the considerable evidence that has been collected regarding the risk factors for the development of lymphedema, the predictive indicators for severe lymphedema remain poorly understood. Therefore, this study examined, both individually and in combination, intraoperative or preoperative characteristics in BCRL patients in order to identify factors related to severe lymphedema and to develop a nomogram for assessing the severity of the lymphedema within 3 years of surgery. The results of this study revealed that advanced age, IPNs dissection, positive lymph nodes, and low education level were associated with an increased risk of developing severe lymphedema. IPNs dissection, in particular, had a greater influence on the development of severe lymphedema than the other risk factors. The nomogram, which integrated age, IPNs dissection, positive lymph nodes, and education level, showed a moderate performance in predicting the severity of lymphedema three years following surgery. Thus, this nomogram can be considered a useful tool for risk assessment and triage of the severity of lymphedema within 3 years of surgery.

Recent research highlighted a series of risk factors associated with lymphedema, including ALND, BMI, radiotherapy, age, chemotherapy, and the number of cycles of chemotherapy [15, 24, 25]. Chemotherapy may influence the inflammatory response and immunological status of cancer survivors, resulting in negative impacts on the normal operation of the lymphatic system [26]. It was suggetsed that BMI at BCRL diagnosis was the major risk factor associated with severe lymphedema [27]. However, findings from this study showed no significant association between BMI, chemotherapy, the number of cycles of chemotherapy and the severity of lymphedema. We hypothesize that sustained risk factors may determine the risk of developing severe lymph edema, since the negative impacts of BMI, radiotherapy, and chemotherapy can be reversed in subsequent in-hospital treatments, whereas age, educational level, and impaired lymphatic system are irreversible. A meta-analysis of 7 studies depicted that older patients were prone to suffer from severe forms of BCRL, yet the exact cause and effect relationship remains unclear [28]. In has been suggested that impaired contractility, increased permeability and immune cell dysfunction related to aging may explain the faster deterioration seen in older women [29]. Furthermore, this study also highlighted the importance of exploring a patient’s understanding of their disease and treatment options, as lower education levels have been linked to higher risk of severe lymphedema. Previously, Fu et al. demonstrated that BCRL patients with a lower education level were more likely to develop severe symptoms at 12 months post-surgery [30, 31].

It has been demonstrated that the incidence of lymphedema was highest in patients with thirty or more lymph nodes removed and increased with the number of positive nodes [21, 24, 32]. However, the association between the number of positive lymph nodes and the development of severe lymphedema remains unclear. Our study revealed that a larger number of positive nodes contribute to a higher risk of severe lymphedema, even though we did not find an association between total lymph node dissection and severe lymphedema. Generally, the more lymph nodes that are dissected, the more severely impaired lymph flow is. Nevertheless, it appears to be related to the location of the lymph nodes, as evidenced by the contribution of IPN to lymphedema. Thus, further investigations are necessary to delineate the exact role that the number of lymph nodes plays in the development of severe lymphedema. It is possible that lymphatic blockage from tumoral infiltration of the lymph node may be responsible for the slight increase in the risk of severe lymphedema by positive lymph nodes.

Notably, our data revealed that IPNs dissection had a considerable influence on the severity of lymphedema, which had not been addressed by previous studies focusing on BCRL risk factors. These findings suggest that IPNs dissection may be a crucial independent risk factor for the onset of lymphedema, which warrants further validation. IPNs, also referred to as Rotter’s nodes, located between pectoralis major and minor, are one of the lymphatic drainage pathways in breast cancer with a metastatic rate of only 4-9.9% [33]. At present, there is an ongoing discussion regarding whether routinely conducting IPNs dissection has any prospective prognostic or therapeutic benefits despite the recommendation from National Comprehensive Cancer Network (NCCN) in their guidelines [34]. Removal of interpectoral lymphatic tissue can lead to injury of the pectoral nerves and vessels, resulting in muscle atrophy and shoulder pain [35]. Our data further showed the contribution of IPNs removal to the development of severe lymphedema. Therefore, these data suggest that greater importance should be placed on IPN dissection and its inclusion in postoperative lymphatic management. For patients with pN0/N1 breast cancer, IPN clearance can be safely omitted even when modified radical mastectomy (MRM) breast conservation surgery is performed [36].

Our study has several limitations that should be considered. Firstly, our identification of risk factors of severe lymphedema was based on an limited regional retrospective database, which may not take into account potential ethnic or regional differences. Secondly, the internal validation of the results, which was conducted using bootstrapping, was limited by the small number of cases in the retrospective cohort. It would be preferable to validate the findings in external cohorts or prospective cohorts. Thirdly, our study only looked into the contribution of intraoperative clinical and laboratory indicators to severe lymphedema, while postoperative prevention and intervention are essential to the occurrence and development of the condition. Finally, due to the retrospective nature of our study, some risk factors associated with lymphedema were likely not included due to incomplete/unavailable data, which could limit the applicability of the nomogram. Thus, further research is required to combine the intraoperative and postoperative indicators to predict the development of severe lymphedema. Despite these limitations, we believe that the findings of this study are usable in terms of severe lymphedema prevention and further intervention globally.

## Conclusion

In summary, we determined that age, IPNs dissection, positive lymph nodes, and education level were independent risk factors of severe lymphedema, with IPNs dissection having the most significant effect on the development of severe BCRL. These findings could be of use for the formulation of lymphedema surveillance strategies and the instruction of patients in clinical practice. Additionally, a nomogram incorporating age, IPNs dissection, positive lymph nodes, and education level was devised, which may be applied to conveniently evaluate the risk of severe lymphedema in patients undergoing surgery with ALND. Notwithstanding, further investigations in larger, multi-center, and prospective cohorts are necessary to validate these findings.

## Data Availability

All data generated or analyzed during this study are included in this published article.

## Abbreviations

BCRL: Breast cancer-related lymphedema
ALND: axillary lymph node dissection
BMI: body mass index
IPNs: interpectoral lymph nodes
ER: estrogen receptor
PR: progesterone receptor
HER2: human epidermal growth factor receptor 2
OR: odd ratios
C-index: concordance index
NCCN: National Comprehensive Cancer Network
MRM: modified radical mastectomy
